# Supplementary material for: Plastic bending in a semiconducting coordination polymer crystal enabled by delamination
Source: Nat Commun. 2022 Nov 4;13:6645. doi: 10.1038/s41467-022-34351-0 (PMC9636129; doi:10.1038/s41467-022-34351-0)
Supplement: Supplementary file 1 — Supplementary Information [file 41467_2022_34351_MOESM1_ESM.pdf]

Supplementary Information

Plastic bending in a semiconducting coordination polymer crystal enabled by delamination

An *et al.*

Table of Contents

Section 1. Supplementary Figures S1

Section 2. Supplementary Tables S18

Section 3. Supplementary Notes S23

Supplementary References S25

Section 1. Supplementary Figures

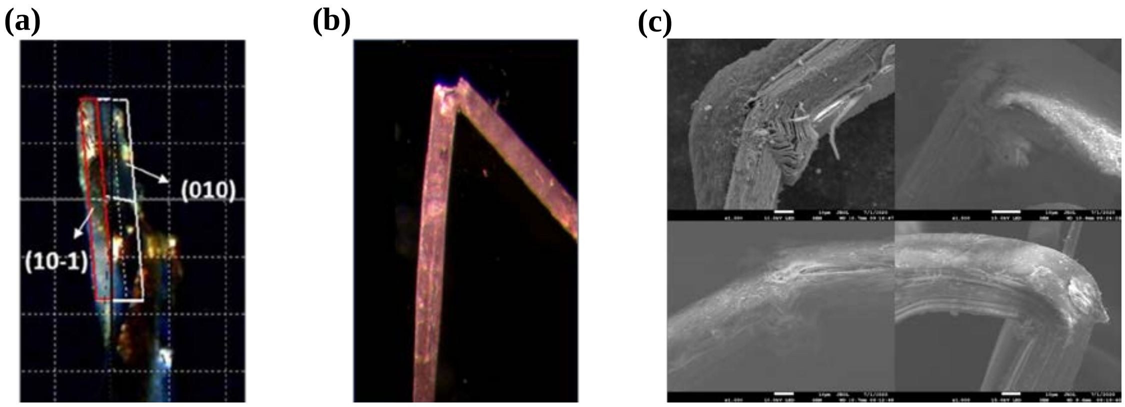

Supplementary Fig. 1 | The face indexation of a Cu-Trz crystal (**a**) and the microscopic images of the crystal bent on the (10-1) face (**b**) and SEM images collected from 4 individually bent crystals (**c**).

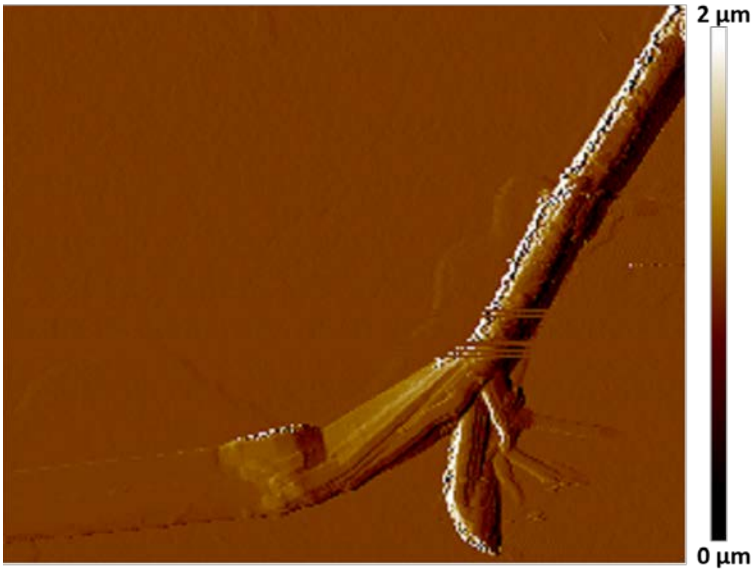

Supplementary Fig. 2 | The AFM image of a bent Cu-Trz crystal.

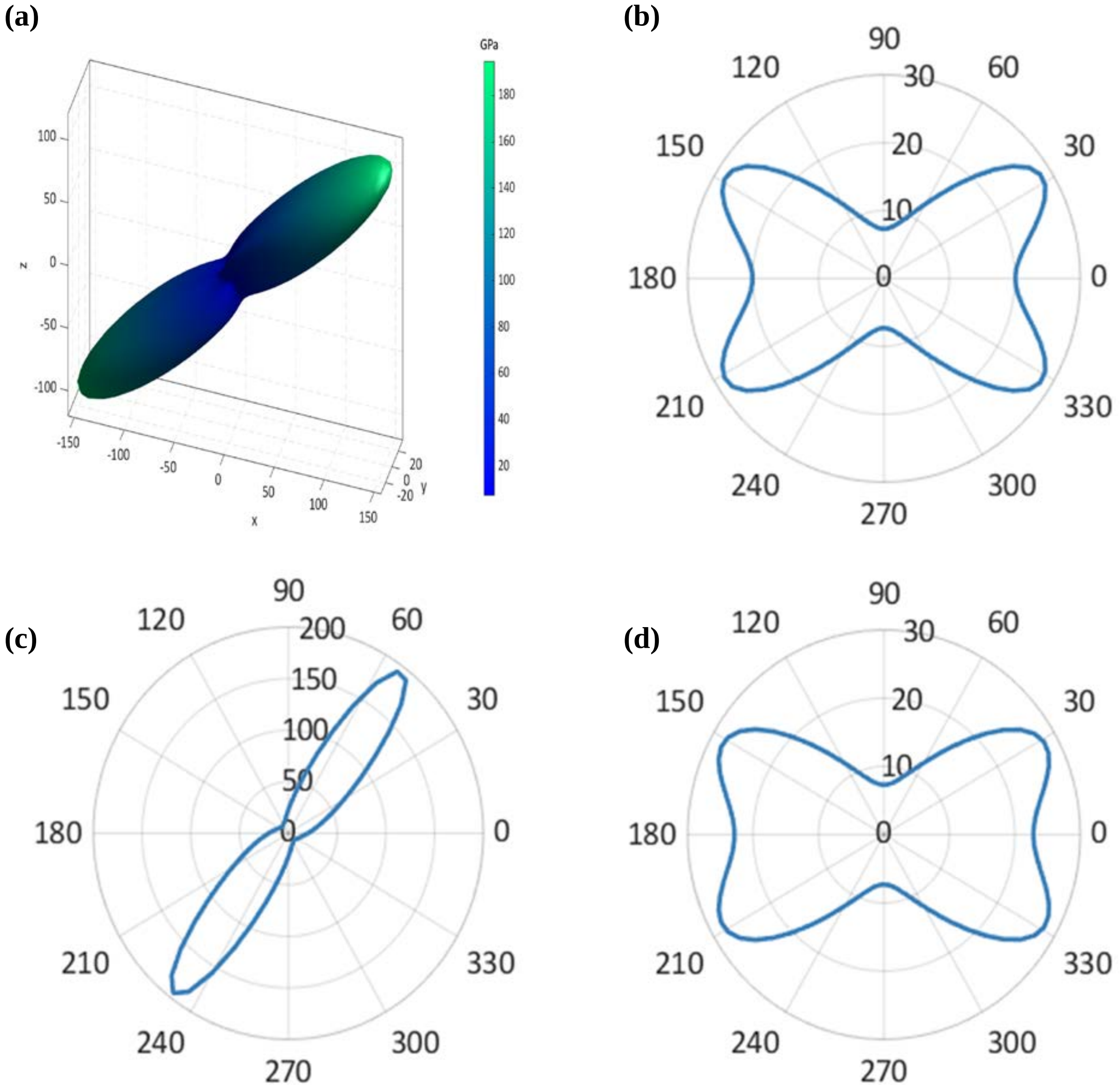

Supplementary Fig. 3 │ Young's moduli of Cu-Trz. (**a**) 3D representation; (**b-d**) 2D representations projected normal to the (100), (010) and (001) planes, respectively.

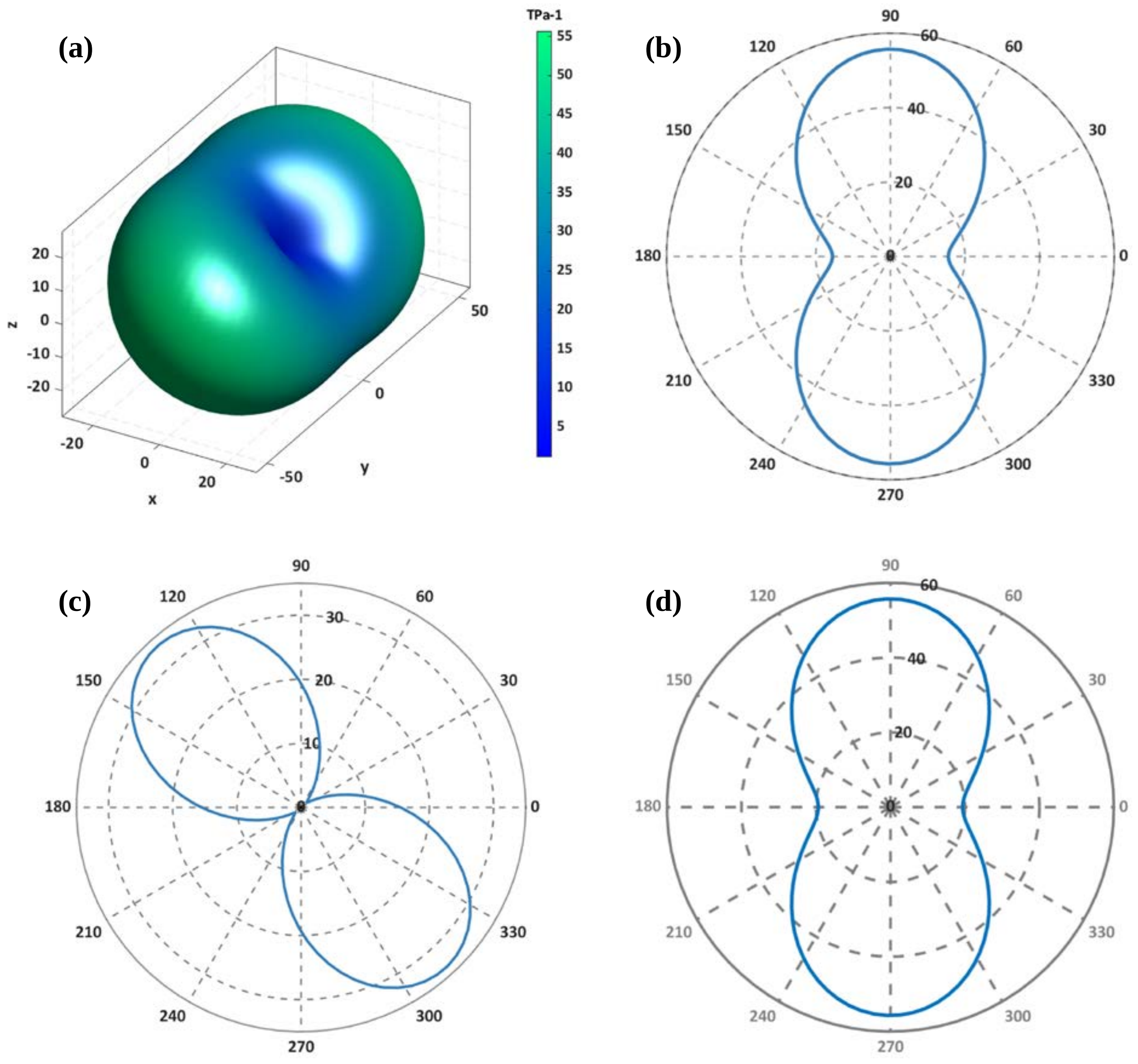

Supplementary Fig. 4 │ Linear compressibilities of Cu-Trz. (**a**) 3D representation; (**b-d**) 2D representations projected normal to the (100), (010) and (001) planes, respectively.

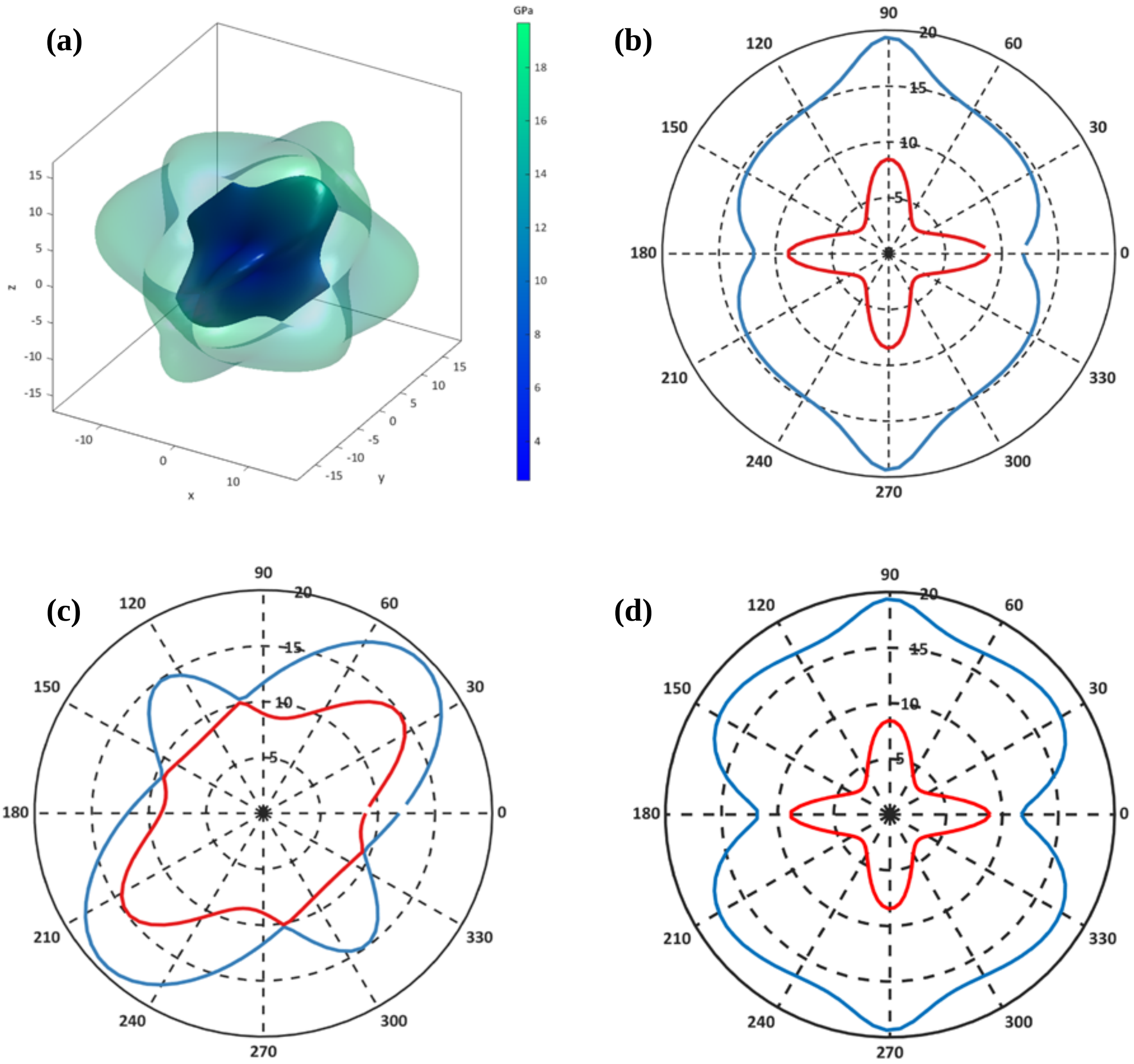

Supplementary Fig. 5 │ Shear moduli of Cu-Trz. (**a**) 3D representation; (**b-d**) 2D representations projected normal to the (100), (010) and (001) planes, respectively.

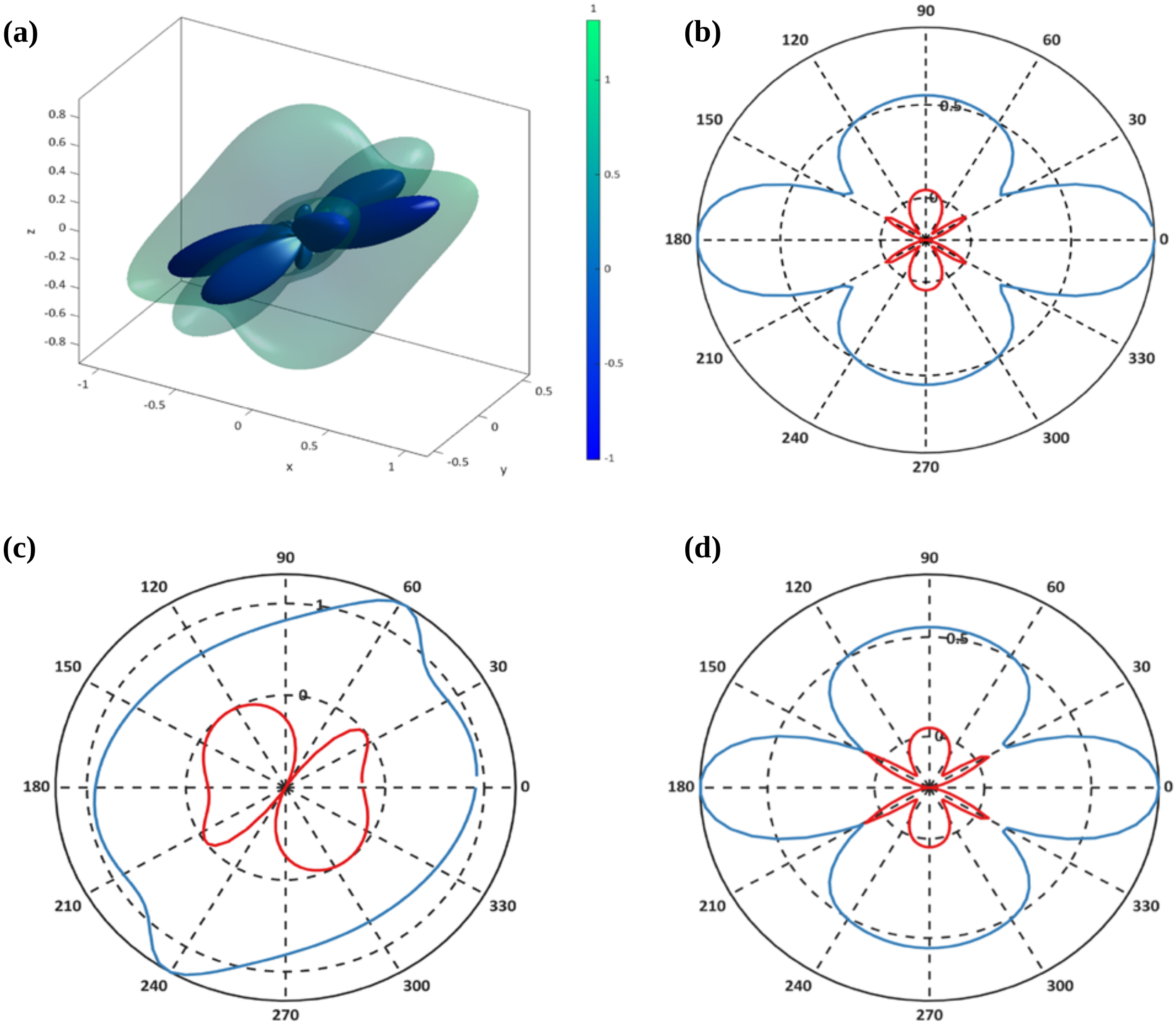

Supplementary Fig. 6 │The Poisson's ratios of Cu-Trz. (**a**) 3D representations; (**b-d**) 2D representations projected normal to the (100), (010) and (001) planes, respectively.

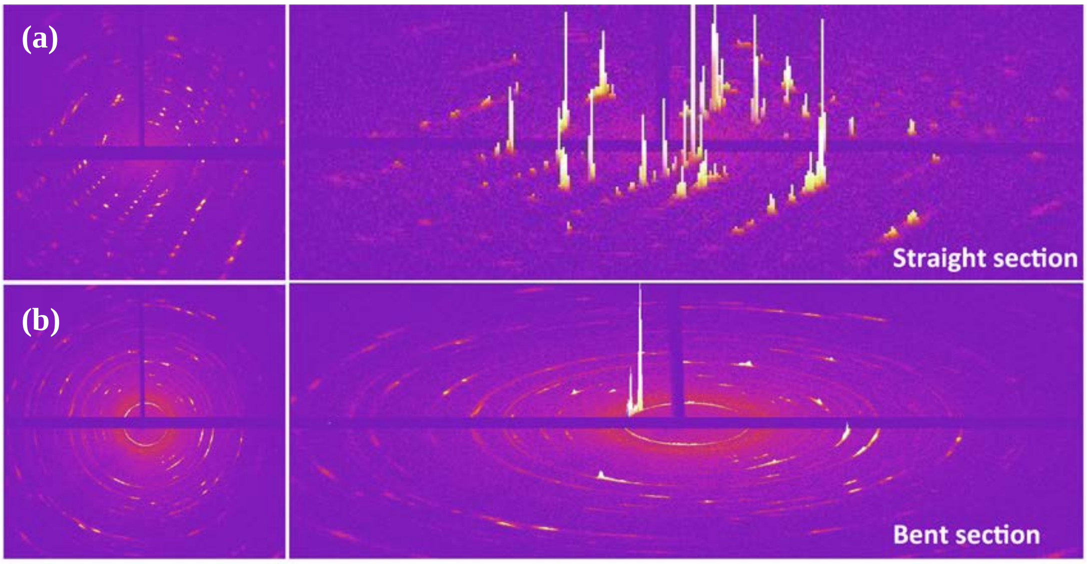

Supplementary Fig. 7 │ 2D diffraction images and 3D views of the straight sections and the bent sections.

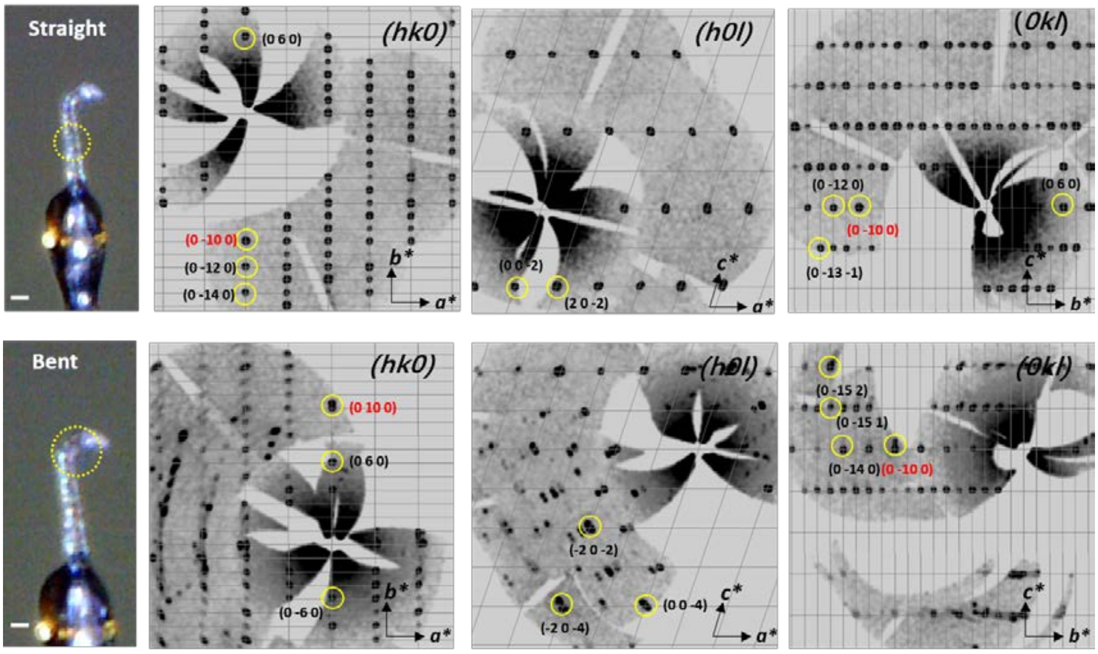

Supplementary Fig. 8 │ The diffraction patterns of straight (top) and bent (bottom) sections collected from a lab facility. The reciprocal spaces are reconstructed based on the X-ray diffraction frames in the (*hk*0), (*h*0*l*), and (0*kl*) planes. The insert scale bar: 0.1 mm.

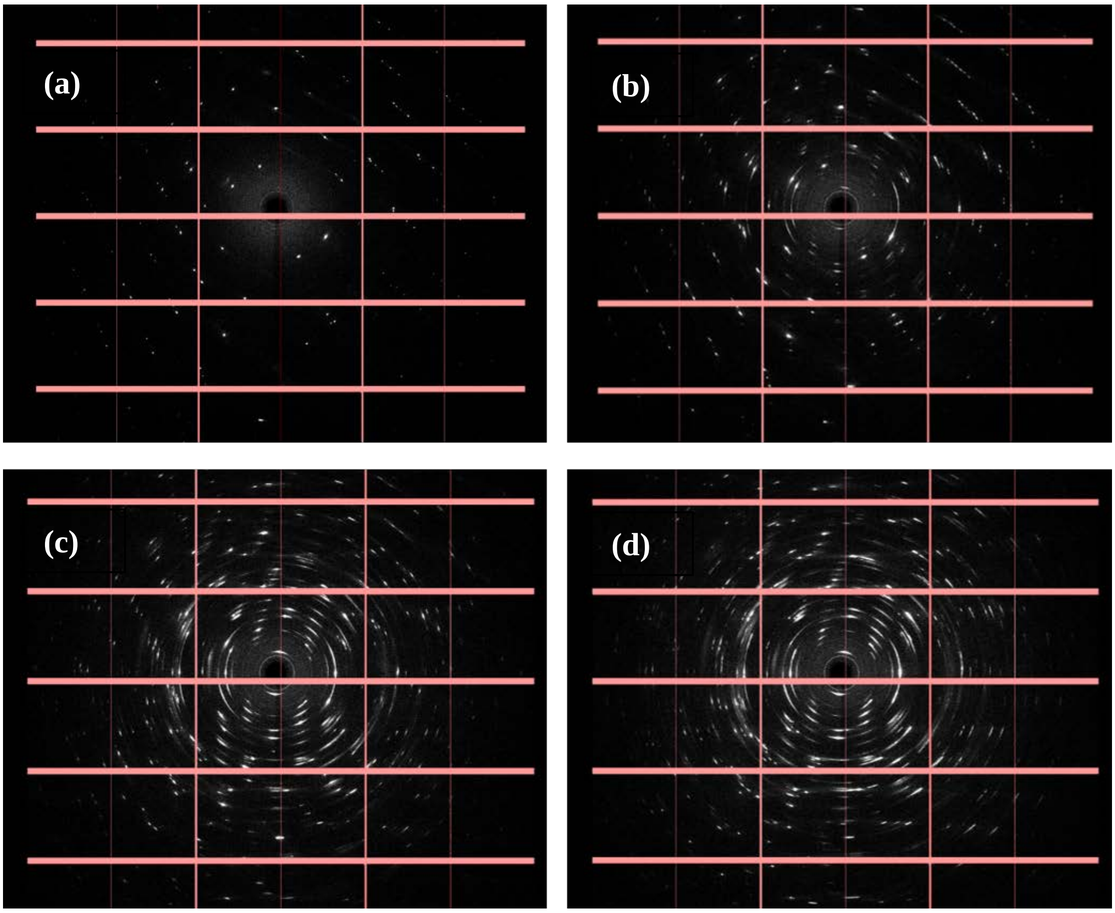

Supplementary Fig. 9 │ Synchrotron diffraction patterns of different regions of a bent Cu-Trz crystal. (**a**) The obtained diffraction pattern of the straight section. (**b-d**) Diffraction patterns of the inner, middle, and outer regions of the bent section.

(a)

(b)

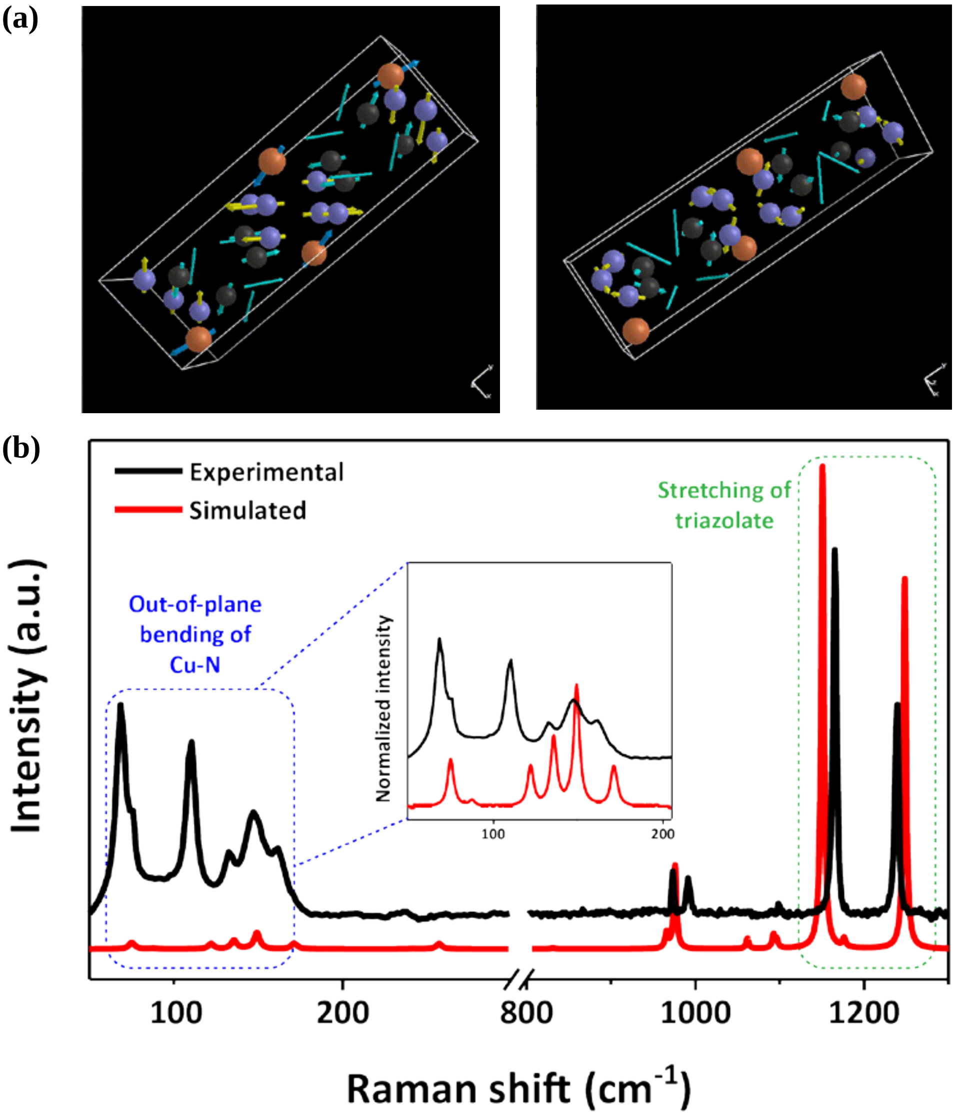

Supplementary Fig. 10 │(**a**) The Raman vibrational modes calculated using DFT; (**b**) Experimental (from crystal #3) and calculated Raman spectra of Cu-Trz. The inset shows the normalized experimental and calculated Raman bands at the low frequency.

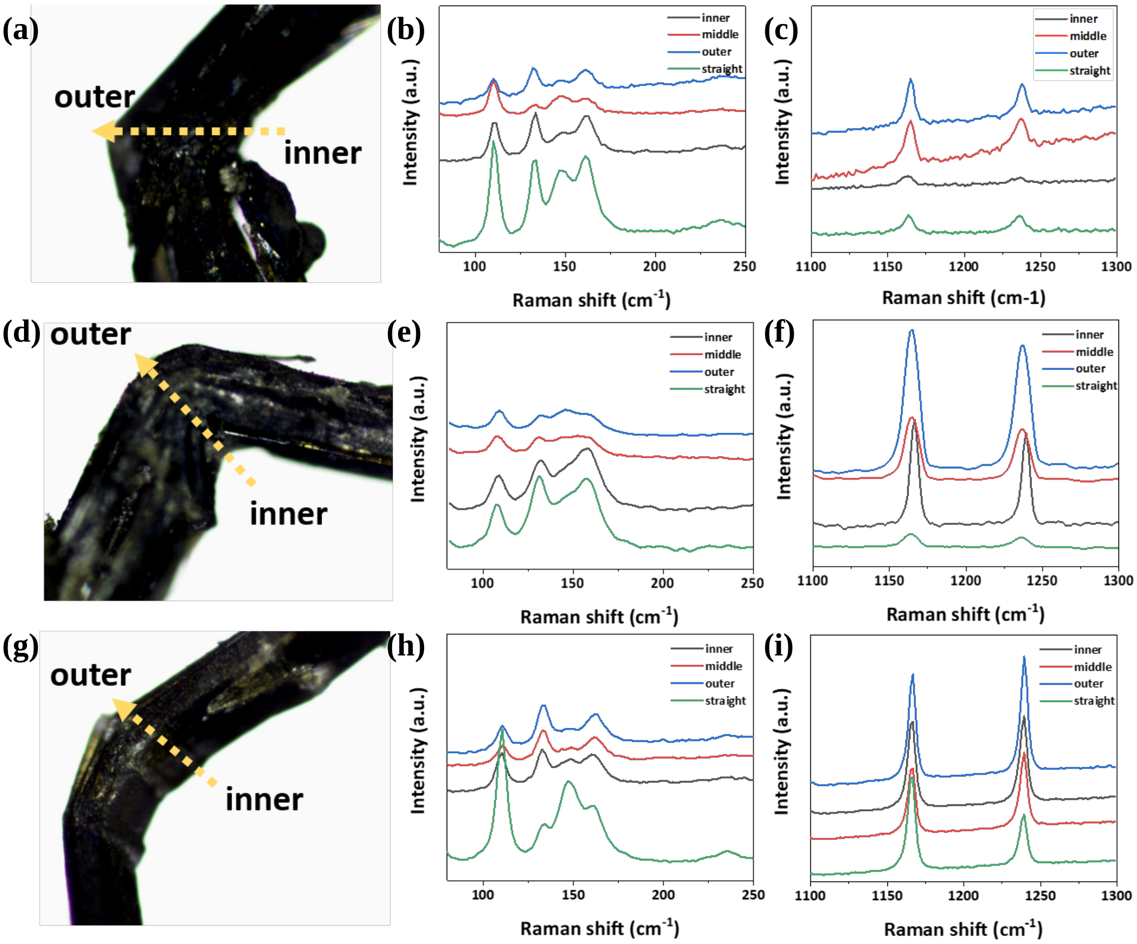

Supplementary Fig. 11 │ The collected Raman spectra from 3 different Cu-Trz crystals. The optical micrographs and the low-wavenumber and high-wavenumber Raman spectra for crystal #1 (**a-c**), crystal #2 (**d-f**), and crystal #3 (**g-i**).

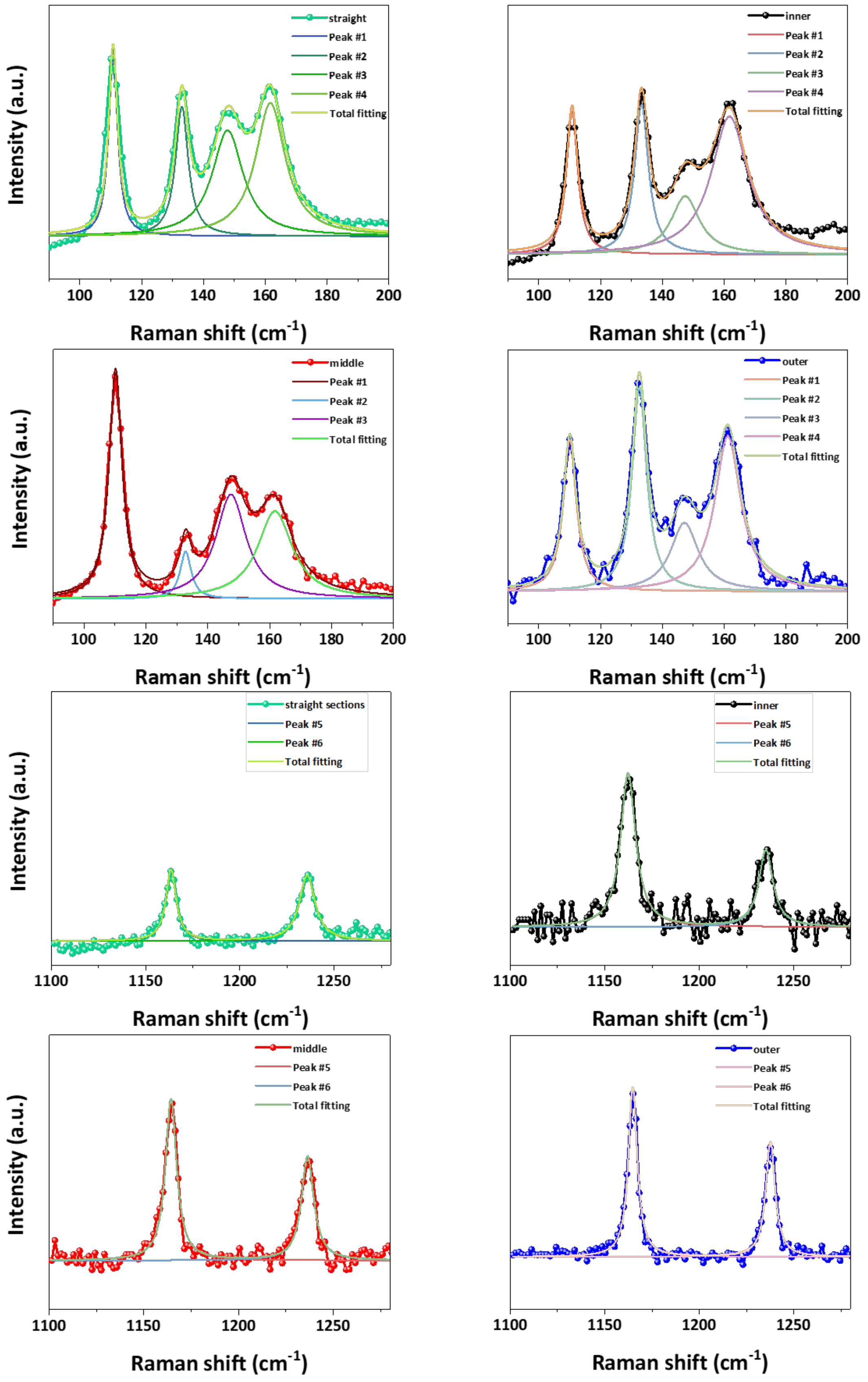

Supplementary Fig. 12 │ The Lorentzian fitting of the Raman bands collected from crystal #1.

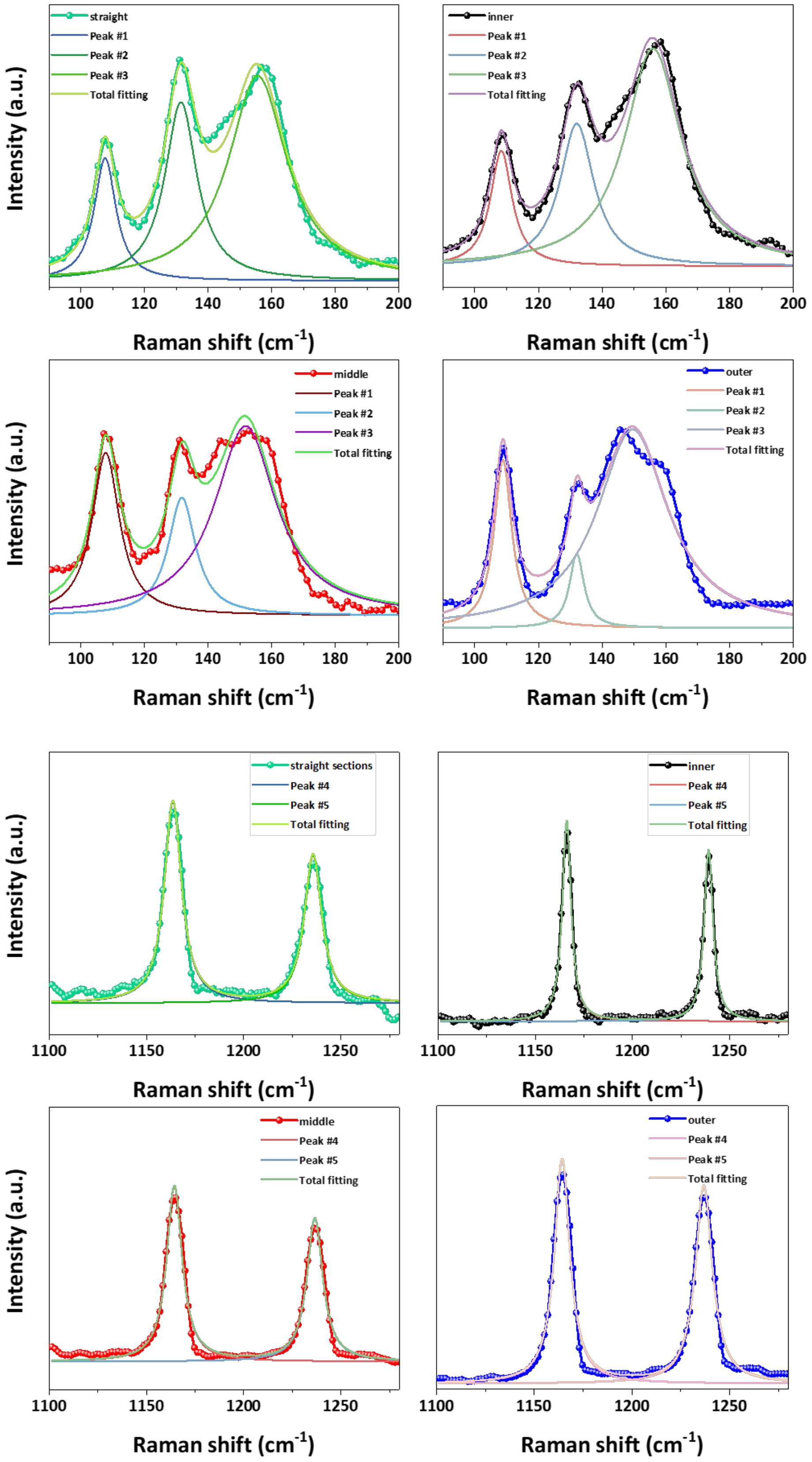

Supplementary Fig. 13 | The Lorentzian fitting of the Raman bands collected from crystal #2.

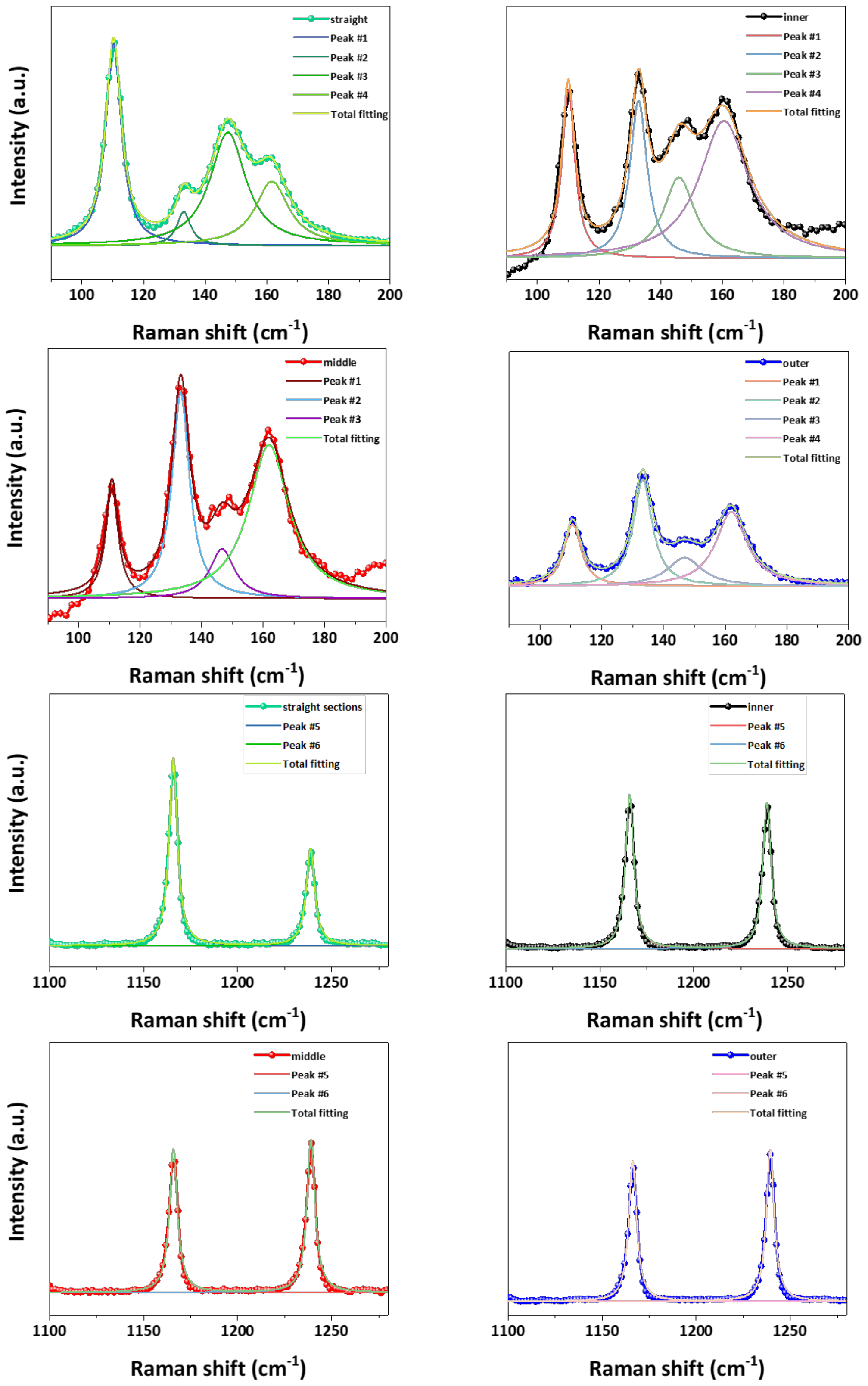

Supplementary Fig. 14 | The Lorentzian fitting of the Raman bands collected from crystal #3.

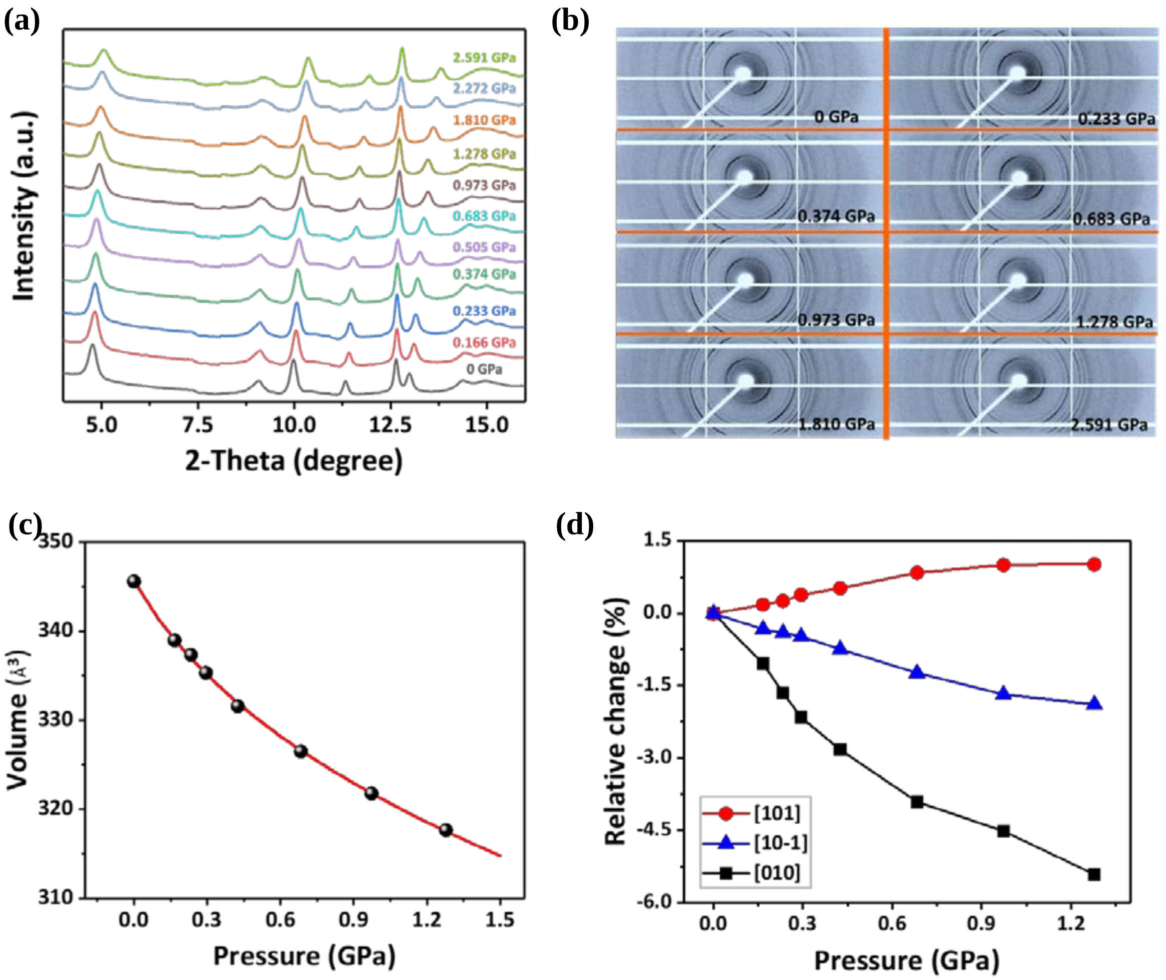

Supplementary Fig. 15 │ The high-pressure response performance of Cu-Trz. (**a**) The HP-PXRD patterns of Cu-Trz collected in the pressure range of 0-2.591 GPa. (**b**) Representative 2D PXRD images at selected pressures. (**c**) The unit cell volume of Cu-Trz crystal as a function of pressure. The red solid line is the third-order Birch–Murnaghan fitting obtained using the PASCal software. (**d**) The relative changes of the lattice parameters of Cu-Trz crystal in dependence of pressure.

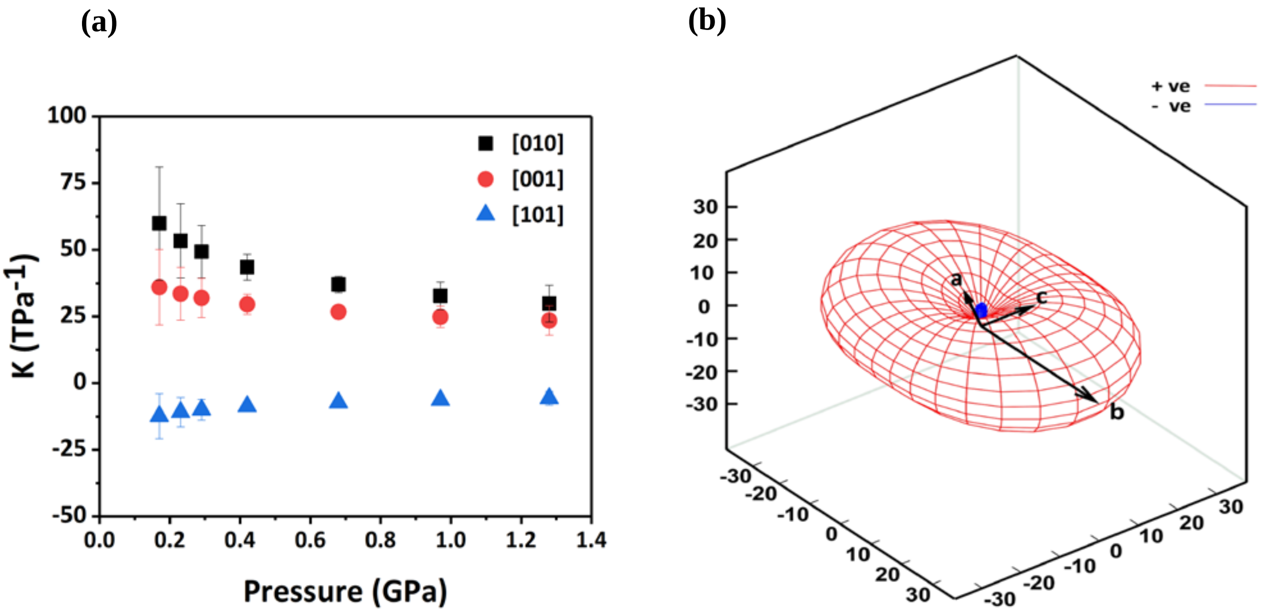

Supplementary Fig. 16 | (**a**) The linear compressibilities along different axes of Cu-Trz extracted from experimental cell parameters. (**b**) The linear compressibility indicatrix of Cu-Trz, with positive and negative linear compressibilities shown in red and blue, respectively.

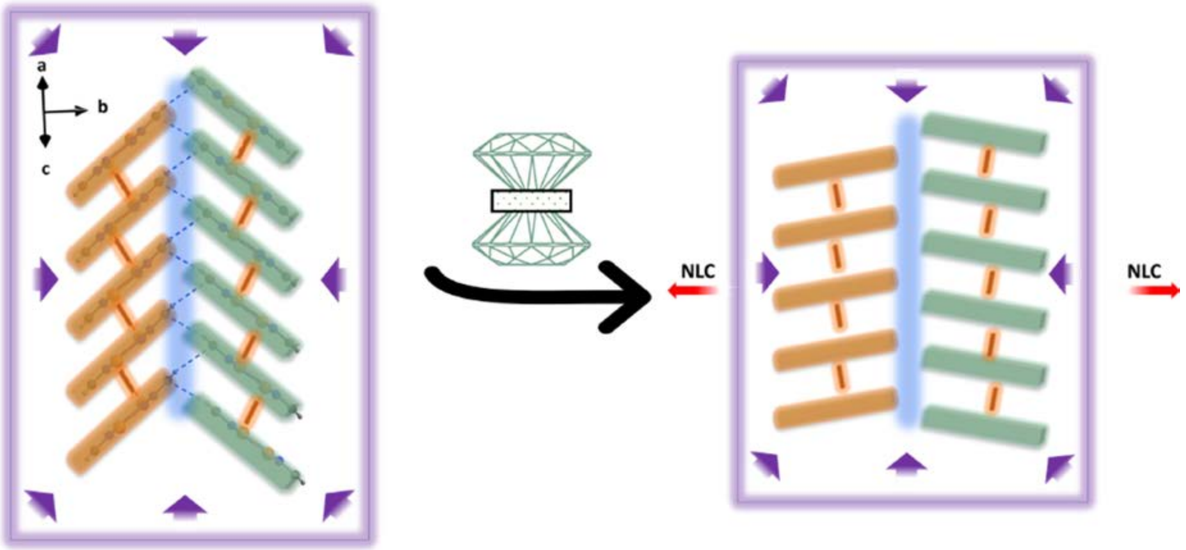

Supplementary Fig. 17 | The schematic mechanisms of the negative linear compressibility in Cu-Trz upon hydrostatic compression.

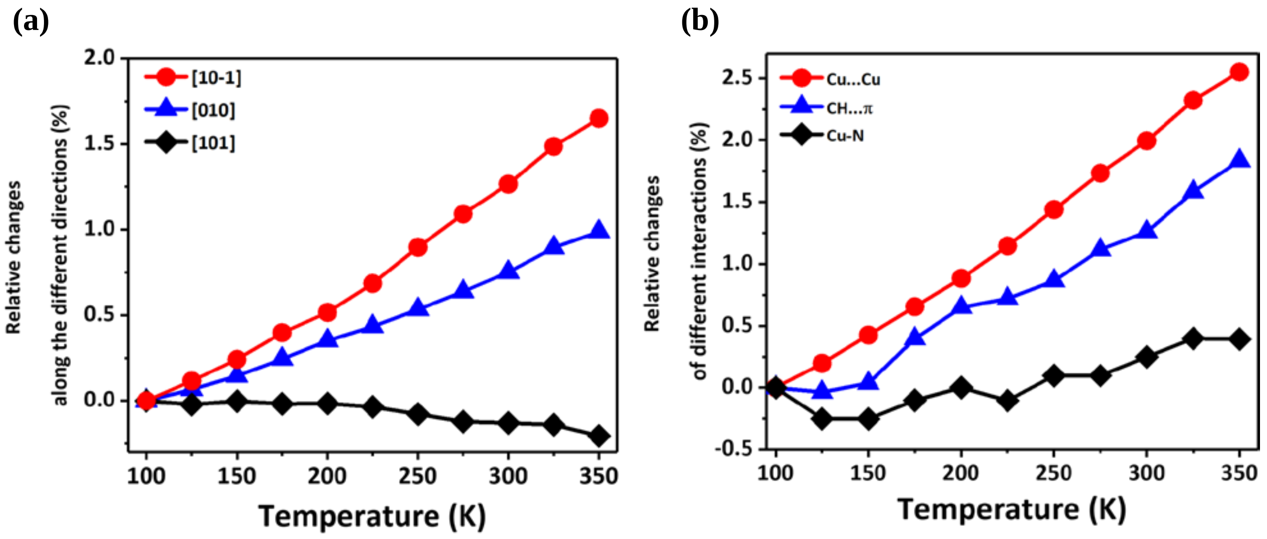

Supplementary Fig. 18 │ The relative changes in different crystallographic directions (**a**) and bonding interactions (**b**) as a function of temperature for Cu-Trz.

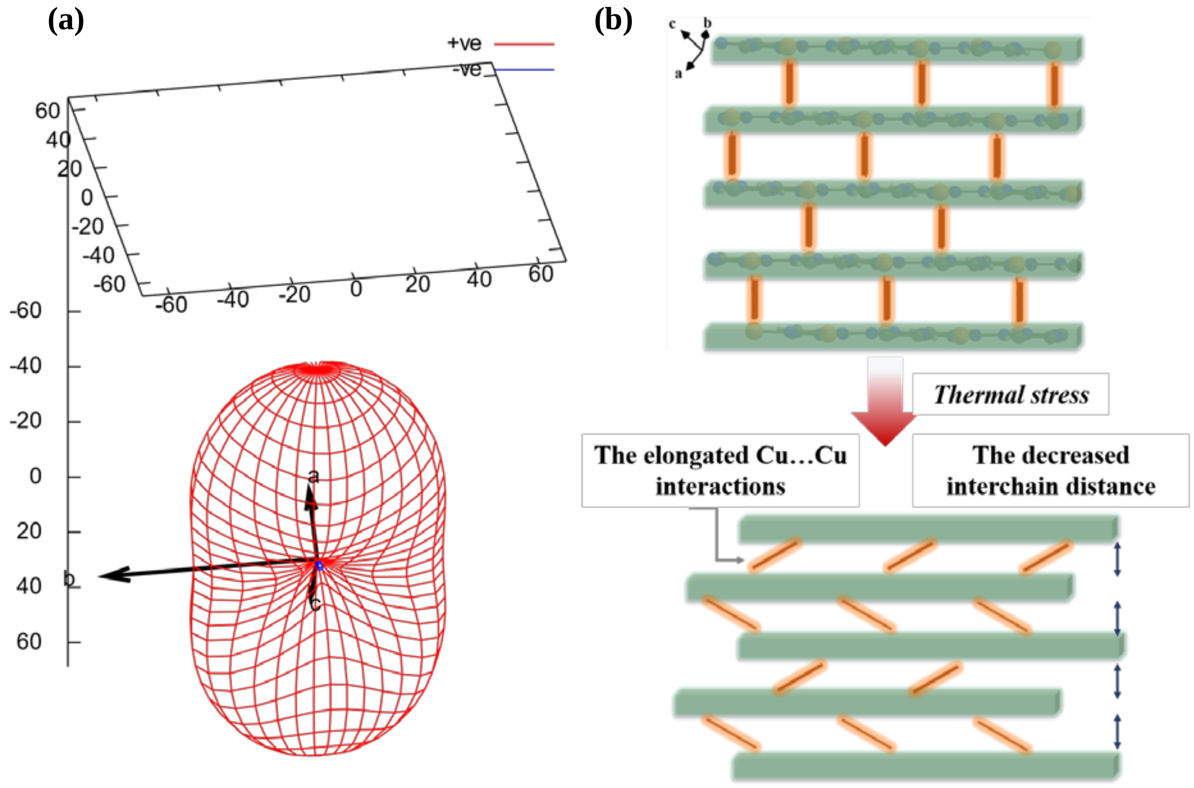

Supplementary Fig. 19 │ **The thermal response performance of Cu-Trz.** (**a**) The thermal expansivity indicatrix of Cu-Trz, with positive and negative thermal expansion in red and blue, respectively. (**b**) The schematic mechanism of negative thermal expansion for Cu-Trz.

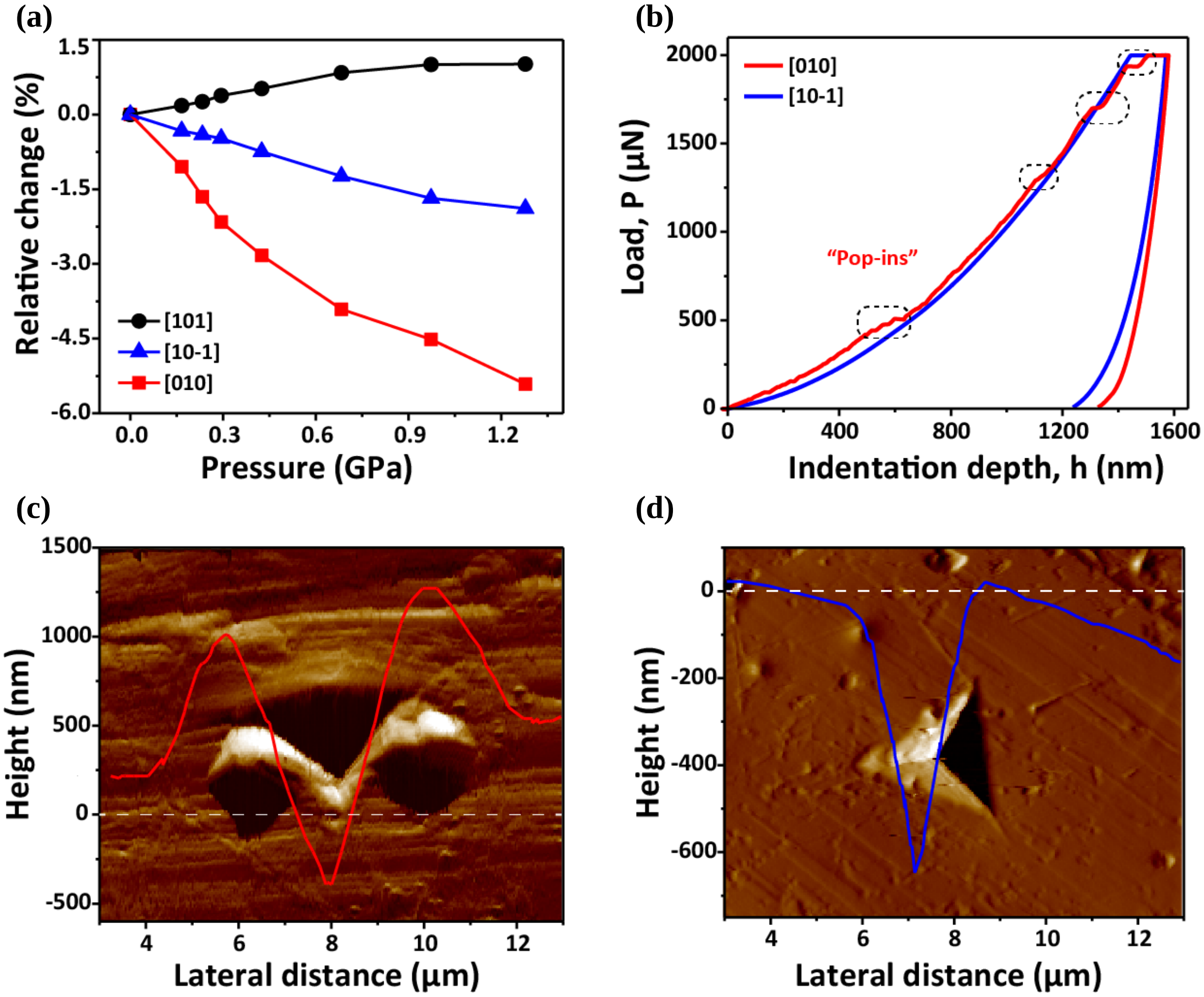

Supplementary Fig. 20 │ The relative changes of different directions under different pressure and the nanoindentation experiments. (**a**) The relative changes along with different crystal directions of Cu-Trz in dependence on pressure. (**b**) The *P*-*h* curves measured normal to the (010) and (10-1) faces of Cu-Trz crystals. Relative height profiles were taken from the scanning probe microscopy (SPM) images (inset) along the [010] **(c)** and [10-1] direction **(d)**.

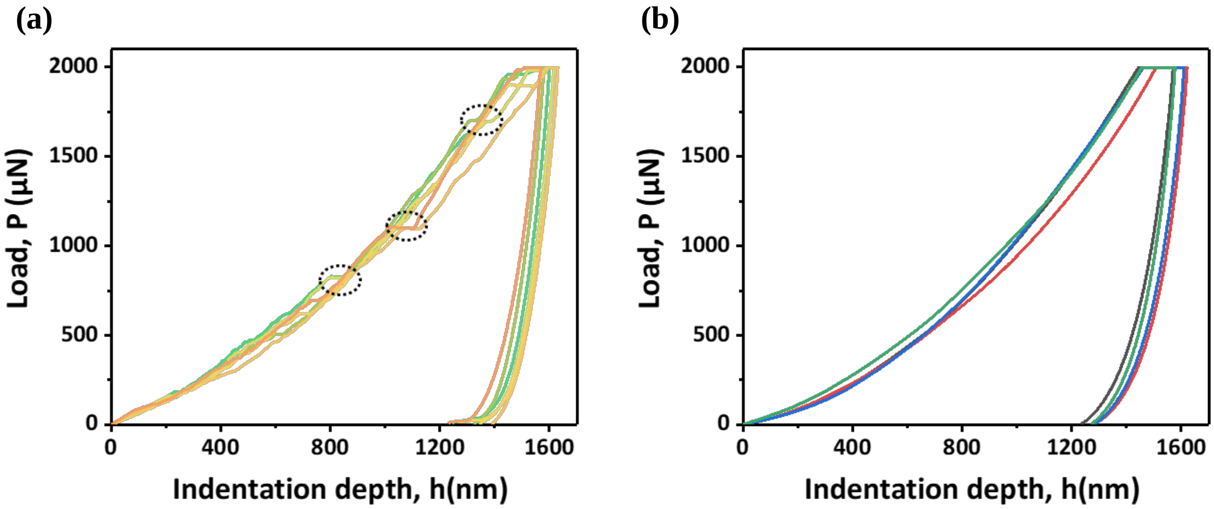

Supplementary Fig. 21 │ The *P*-*h* curves measured from the (010) (**a**) and (10-1) face (**b**) of Cu-Trz crystals.

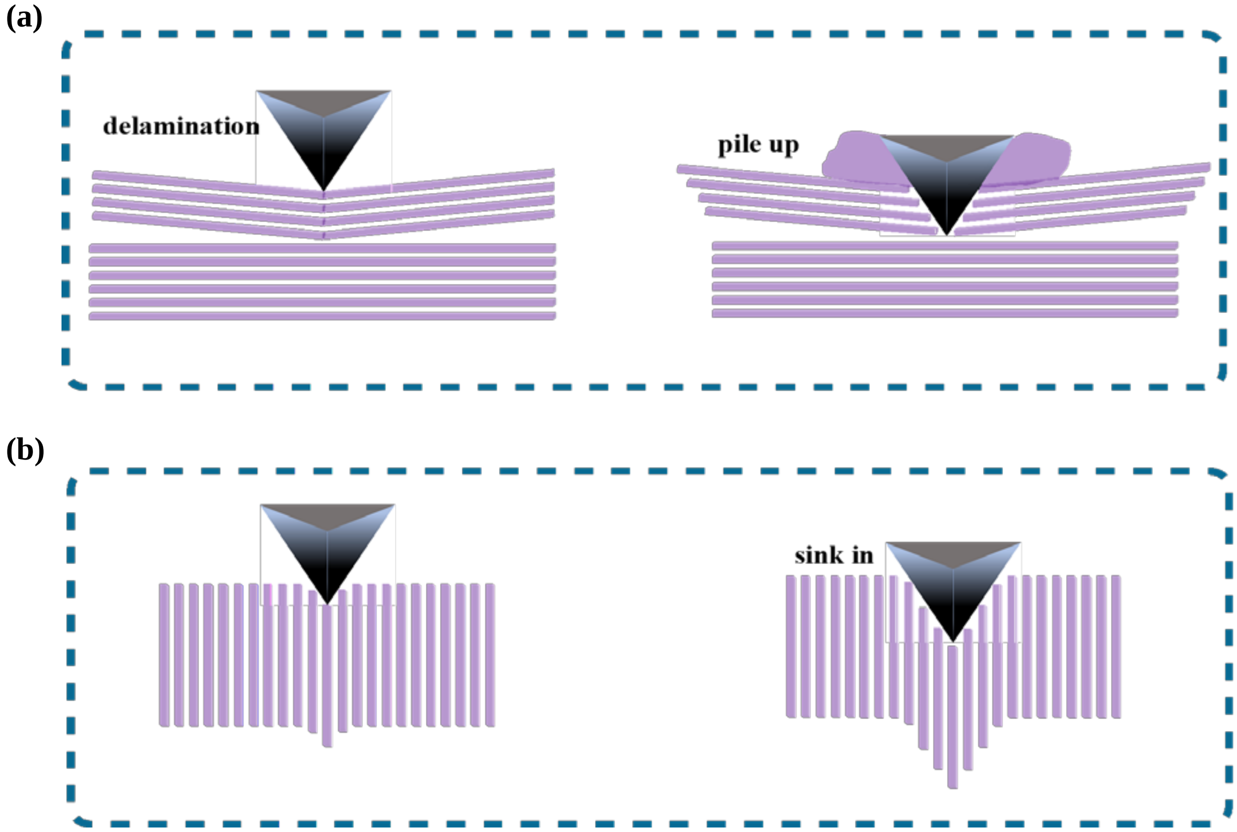

Supplementary Fig. 22 │ The proposed mechanisms for different indentation processes normal to the (010) (**a**) and (10-1) (**b**) faces.

Section 2. Supplementary Tables

Supplementary Table 1 │ The elastic tensors of Cu-Trz crystal obtained from DFT calculations.

$$\begin{pmatrix} 96.94 & 8.11 & 37.81 & 0 & 59.27 & 0 \\ 8.11 & 12.56 & 9.94 & 0 & 0.32 & 0 \\ 37.81 & 9.94 & 49.31 & 0 & 32.52 & 0 \\ 0 & 0 & 0 & 14.03 & 0 & 5.46 \\ 59.27 & 0.32 & 32.52 & 0 & 51.70 & 0 \\ 0 & 0 & 0 & 5.46 & 0 & 13.81 \end{pmatrix}$$

Supplementary Table 2 │ Summary of elastic properties of Cu-Trz crystal. The anisotropies are denoted by $A_X = X_{max}/X_{min}$.

Properties		Values	Directions	Anisotropies (X_{max} / X_{min})	Bulk modulus (K, GPa)
Young's moduli (E, GPa)	E_{max}	194.93	<101>	26.71	
	E_{min}	7.30	<010>		
Shear moduli (G, GPa)	G_{max}	19.39	(101) <010>	7.66	
	G_{min}	2.53	(11-1) <-111>		11.04 ± 2.01
Poisson's ratios (ν, TPa^{-1})	ν_{max}	1.32	<201, 010>	∞	
	ν_{min}	-1.01	<201, -102>		
Compressibilities (β, GPa)	β_{max}	55.71	<010>	47.46	
	β_{min}	1.17	<101>		

Supplementary Table 3 │ The peak positions and FWHMs obtained by the Lorentzian fitting of Raman data collected from crystal #1.

**Peak position (cm^{-1})**	Straight	inner	middle	outer
Peak #1	110.8	110.8	110.2	110.0
Peak #2	133.0	133.3	132.8	132.6
Peak #3	147.8	147.4	147.5	147.2
Peak #4	161.7	161.8	161.7	161.2
Peak #5	1163.5	1162.3	1164.4	1164.8
Peak #6	1235.7	1235.3	1236.5	1237.7

**FWHM (cm^{-1})**	Straight	inner	middle	outer
Peak #1	3.5	4.4	5.1	5.2
Peak #2	5.0	5.5	4.3	5.7
Peak #3	12.2	11.1	11.9	11.0
Peak #4	11.7	14.4	13.7	10.2
Peak #5	6.6	9.6	7.2	5.7
Peak #6	10.1	9.2	8.0	6.1

Supplementary Table 4 │ The peak positions and FWHMs obtained by the Lorentzian fitting of Raman data collected from crystal #2.

**Peak position (cm^{-1})**	Straight	inner	middle	outer
Peak #1	107.6	108.3	107.8	108.8
Peak #2	131.4	132.0	131.7	132.0
Peak #3	155.6	155.9	151.8	150.5
Peak #4	1163.6	1166.2	1166.4	1164.3
Peak #5	1234.7	1239.1	1236.6	1236.9

**FWHM (cm^{-1})**	Straight	inner	middle	outer
Peak #1	7.2	8.3	10.5	6.8
Peak #2	12.2	13.0	10.5	5.6
Peak #3	20.2	21.5	23.5	27.0
Peak #4	10.7	5.5	9.7	9.8
Peak #5	10.8	5.5	10.0	10.7

Supplementary Table 5 │The peak positions and FWHMs obtained by the Lorentzian fitting of Raman data collected from crystal #3.

**Peak position (cm^{-1})**	Straight	inner	middle	outer
Peak #1	110.3	110.0	110.8	110.6
Peak #2	133.0	132.8	133.2	133.3
Peak #3	147.4	146.0	146.6	146.9
Peak #4	161.6	160.5	161.9	162.0
Peak #5	1165.8	1165.9	1165.9	1166.2
Peak #6	1238.7	1238.9	1239.0	1239.4

**FWHM (cm^{-1})**	Straight	inner	middle	outer
Peak #1	5.2	5.1	5.0	6.7
Peak #2	5.5	6.8	6.9	7.5
Peak #3	12.6	12.4	9.7	13.8
Peak #4	12.1	19.4	16.1	12.3
Peak #5	5.3	5.3	5.2	5.1
Peak #6	5.5	5.3	5.2	5.0

Supplementary Table 6 │Cell parameters extracted from HP-PXRD experiments.

**Pressure (GPa)**	***a*** **(Å)**	***b*** **(Å)**	***c*** **(Å)**	**β (°)**	***V*** **($Å^3$)**
0	4.873	14.847	5.053	109.562	345.559
0.166	4.876	14.691	5.028	109.364	338.948
0.233	4.879	14.602	5.018	109.337	337.324
0.294	4.884	14.526	5.008	109.295	335.305
0.425	4.888	14.426	4.979	109.226	331.577
0.683	4.898	14.266	4.932	109.021	326.495
0.973	4.899	14.176	4.900	108.792	321.757
1.278	4.901	14.043	4.877	108.825	317.650

Supplementary Table 7 │ Selected bond distances of Cu-Trz as a function of temperature.

**Temperature (K)**	**Cu···Cu (Å)**	**C-H···*π* (Å)**	**Cu-N (Å)**
100	3.057	2.779	1.973
125	3.063	2.778	1.971
150	3.070	2.780	1.971
175	3.077	2.790	1.972
200	3.084	2.797	1.973
225	3.092	2.799	1.972
250	3.101	2.803	1.974
275	3.110	2.810	1.974
300	3.118	2.814	1.975
325	3.128	2.823	1.976
350	3.135	2.830	1.976

Supplementary Table 8 │ Cell parameters of Cu-Trz as a function of temperature.

**Temperature (K)**	***a* (Å)**	***b* (Å)**	***c* (Å)**	**β (°)**	***V* (Å^3)**
100	4.894	14.931	4.978	108.814	344.290
125	4.896	14.940	4.982	108.887	344.838
150	4.900	14.953	4.987	108.942	345.614
175	4.904	14.968	4.992	109.035	346.436
200	4.908	14.984	4.997	109.095	347.229
225	4.912	14.996	5.003	109.196	348.030
250	4.917	15.011	5.010	109.330	348.961
275	4.923	15.027	5.016	109.459	349.851
300	4.928	15.043	5.022	109.556	350.821
325	4.933	15.065	5.031	109.675	352.054
350	4.937	15.079	5.036	109.798	352.720

Supplementary Table 9 │ A comparison between the optimized crystal structure and the one of 100 K.

	the experimental structure of 100 K	the optimized structure
a (Å)	4.894	4.864
b (Å)	14.931	14.581
c (Å)	4.978	5.010
α (°)	90	90
β (°)	108.814	108.803
γ (°)	90	90
V (Å^3)	344.300	336.313

Supplementary Table 10 │ Semiconductive properties for some known coordination polymers.

Material	σ (S/cm)	Reference
Fe(1,2,3-triazolate)$_2$	7×10^{-9}	1
Co(1,2,3-triazolate)$_2$	5.1×10^{-14}	2
Cd(1,2,3-triazolate)$_2$	1.4×10^{-13}	2
Cu(1,2,3-triazolate)$_2$	3.2×10^{-14}	2
Mg(1,2,3-triazolate)$_2$	9.9×10^{-15}	2
Mn(1,2,3-triazolate)$_2$	8.2×10^{-14}	2
Zn(1,2,3-triazolate)$_2$	1.2×10^{-14}	2

Section 3. Supplementary Notes

Supplementary Note 1

The elastic constants (**Supplementary Table 1**) of Cu-Trz were obtained *via* the density functional theory calculations which reveal its significant mechanical anisotropy. As shown in **Supplementary Fig. 3 and Supplementary Table 2**, the maximum of E (E_{max}) is 194.93 GPa along <101> direction (the chain direction) while the minimum of E (E_{min}) is 7.30 GPa along <010> direction (approximately along the C–H···π interactions), leading to a very large anisotropy (E_{max}/E_{min} = 26.71). Meanwhile, the value of E along <10-1> direction, approximate direction of Cu···Cu interactions, is 9.24 GPa. This value lies in the middle between the E_{max} and E_{min}, indicating that the Cu···Cu interactions possess the intermediate mechanical strength between the Cu–N bonding and the C–H···π interactions. The large anisotropy of Cu-Trz can also be reflected by its linear compressibilities, shear moduli and Poisson's ratios **(Supplementary Figs. 4-6).**

Supplementary Note 2

The high-pressure X-ray powder diffractions (HP-PXRD) (**Supplementary Fig. 15**) reveal that negative linear compressibility (NLC) occurs along the [101] direction (**Supplementary Fig. 16**, **Table 6**). The linear compressibilities of b and c axes are respectively 36.96 and 26.76 TPa^{-1} while the NLC of the [101] direction is -7.38 TPa^{-1}. As shown in **Supplementary Fig. 17**, these flexible supramolecular interactions can produce slippage under increasing hydrostatic pressure and the adaptive changes flatten the herringbone packing motif, consequently leading to NLC along [101].

Supplementary Note 3

Further variable temperature single crystal X-ray diffractions (VT-SCXRD) were carried out to study the response of Cu-Trz to thermal stress. As shown in **Supplementary Fig. 18**, the relative changes of the Cu···Cu and the C–H···π interactions are larger than that of Cu–N bonds, suggesting the anisotropic thermal responses of different bonding forces. The obtained coefficients of thermal expansion (α) are $\alpha_{[10\text{-}1]}$ = 69.9, $\alpha_{[010]}$ = 42.0 and $\alpha_{[101]}$ = -8.7 MK^{-1}. The relatively large coefficients along [10-1] and [010] are ascribed to the weak Cu···Cu and C–H···π interactions which can be easily affected by the thermal stress (**Supplementary Fig. 19a**). Interestingly, there is a negative thermal expansion (NTE, $\alpha_{[101]}$ = -8.7 MK^{-1}) along the Cu–N direction. As shown in **Supplementary Fig. 19b**, the thermal stress can induce pronounced transverse vibrations of Cu atoms which leads to elongated Cu···Cu distance between adjacent chains. Then adjacent chains slide to each other due to the

elongated Cu···Cu interactions, resulting in the decreased interchain distance and corresponding NTE along the [101] direction.

Supplementary Note 4

The representative load-penetration (*P*-*h*) curves (**Supplementary Fig. 20**) of (010) and (10-1) faces exhibit great residual depths from unloading, indicating that great plastic deformation occurred during indentation of both faces. According to the Pharr-Oliver equation, the *E* and *H* values were extracted from *P*-*h* plots (**Supplementary Fig. 21**) as $E_{(010)}$ = 5.29 ± 0.15, $H_{(010)}$ = 0.21 ± 0.01 and $E_{(10\text{-}1)}$ = 5.05 ± 0.27, $H_{(10\text{-}1)}$ = 0.22 ± 0.01 GPa, respectively. The low elastic moduli and hardnesses of both faces suggest the very soft nature of Cu-Trz crystals. During a typical loading segment of the (010) face, typical displacement bursts ("pop-ins") occurred in the *P*-*h* curves, which implies the sudden penetration of the indenter tip at a certain load (**Supplementary Fig. 20b**). These "pop-ins" could be attributed to the stress localization in the (010) face which break the C–H···π interactions between the adjacent "layers" hold by Cu–N bonds and Cu···Cu interactions, leading to the final delamination. Meanwhile, two distinct ridges ("piles-up") along two edges of the indenter imprint on the (010) face were observed after unloading (**Supplementary Fig. 20c**). Such "piles-up" arise from the flowing up of the delaminated structures and reflect the plastic and soft nature of the bent crystals. On the contrary, the loading segment of the *P*-*h* curve of the (10-1) face is smooth, indicating a continuous penetration (**Supplementary Fig. 20d**). As shown in **Supplementary Fig. 22**, there are evident sink-ins instead of piles-up, which reveals that the stress can easily break the intralayer C–H···π interactions and produce a great displacement without any stress localization. The smooth *P*-*h* curves and the sink-ins of (10-1) disclose that the stress cannot be efficiently resolved by the localization during the penetration, hence resulting in the final fracture upon bending.

Supplementary References

1. Park, J. G. et al. Charge delocalization and bulk electronic conductivity in the mixed valence metal−organic framework Fe(1,2,3-triazolate)$_2$(BF$_4$)$_x$. *J. Am. Chem. Soc.* **140**, 8526−8534 (2018).
2. Sun, L. et al. Is iron unique in promoting electrical conductivity in MOFs? *Chem. Sci.* **8**, 4450−4457 (2017).
