# Supplementary material for: Plastic bending in a semiconducting coordination polymer crystal enabled by delamination
Source: Nat Commun. 2022 Nov 4;13:6645. doi: 10.1038/s41467-022-34351-0 (PMC9636129; doi:10.1038/s41467-022-34351-0)
Supplement: Supplementary file 3 — Description of Additional Supplementary Files [file 41467_2022_34351_MOESM3_ESM.pdf]

Description of Additional Supplementary Files

Supplementary Movie 1: The calculated Raman active modes in different wavenumber ranges.
